# Ethylene-Octene-Copolymer with Embedded Carbon and Organic Conductive Nanostructures for Thermoelectric Applications

**DOI:** 10.3390/polym12061316

**Published:** 2020-06-09

**Authors:** Petr Slobodian, Pavel Riha, Robert Olejnik, Michal Sedlacik

**Affiliations:** 1 Centre of Polymer Systems, University Institute, Tomas Bata University, Tr. T. Bati 5678, 760 01 Zlin, Czech Republic; olejnik@utb.cz (R.O.); msedlacik@utb.cz (M.S.); 2 Faculty of Technology, Polymer Centre, Tomas Bata University, T.G.M. 275, 760 01 Zlin, Czech Republic; 3 The Czech Academy of Sciences, Institute of Hydrodynamics, Pod Patankou 5, 166 12 Prague 6, Czech Republic; 4 Department of Production Engineering, Faculty of Technology, Tomas Bata University in Zlin, T. G. Masaryk nam. 275, 762 72 Zlin, Czech Republic

**Keywords:** ethylene-octene-copolymer, carbon nanotubes, carbon fibers, polyaniline, polypyrrole, thermoelectric composites

## Abstract

Hybrid thermoelectric composites consisting of organic ethylene-octene-copolymer matrices (EOC) and embedded inorganic pristine and functionalized multiwalled carbon nanotubes, carbon nanofibers or organic polyaniline and polypyrrole particles were used to form conductive nanostructures with thermoelectric properties, which at the same time had sufficient strength, elasticity, and stability. Oxygen doping of carbon nanotubes increased the concentration of carboxyl and C–O functional groups on the nanotube surfaces and enhanced the thermoelectric power of the respective composites by up to 150%. A thermocouple assembled from EOC composites generated electric current by heat supplied with a mere short touch of the finger. A practical application of this thermocouple was provided by a self-powered vapor sensor, for operation of which an electric current in the range of microvolts sufficed, and was readily induced by (waste) heat. The heat-induced energy ensured the functioning of this novel sensor device, which converted chemical signals elicited by the presence of heptane vapors to the electrical domain through the resistance changes of the comprising EOC composites.

## 1. Introduction

Thermoelectric conductive polymer composites convert thermal energy into electricity when there is a difference in temperatures between the hot and cold junctions of such two dissimilar conductive or semiconductive composites. The conversion is based on a phenomenon called the Seebeck effect. The heat supplied at the hot junction (hot side) of the thermoelectrics causes a flow of electric current to the cold side that can be harnessed as useful voltage.

The classical thermoelectrics are made from inorganic materials such as metals (Al, Cu, Ni), metallic alloys (chromel, alumel) and semiconductors (PbT, Bi_2_Te_3_), which are thermoelectrically efficient, but at the same time expensive, heavy, and some of them materially in short supply. Consequently, alternative organic thermoelectrics are being developed. These organic thermoelectrics do not produce as much energy as the metal ones. The thermoelectric figure of merit is typically only in the range 0.001–0.01 at room temperature. However, the mechanical flexibility, processability, light weight, and low manufacturing costs are attractive features for potential applications for electricity microgeneration e.g., in sensors and electronics. 

Petsagkourakis et al. [1] thoroughly reviewed principles and advances in the development of those thermoelectric materials. The electronic properties of the conductive (conjugated) polymers are based on their macromolecular structure and morphology, since they affect the electronic conditions, and therefore the charge transport and the thermoelectric properties. Such conductive polymers have been used mostly for thermoelectric generators and temperature sensors. Hybridizing of such polymers with inorganic thermoelectric materials is another way to enhance their thermoelectric performance. Such hybrids have low thermal conductivity, which is advantageous in energy harvesting. Among others, metals, Bi_2_Te_3_ or carbon nanoparticles have been used as the inorganic portions of such composites.

Polyaniline (PANI) and polypyrrole (PPy) are two of the most studied conductive polymers owing to their easy and low-cost synthesis, good environmental stability, and simple doping/dedoping processes based on acid–base reactions [2,3]. The PANI conductivity apparently depends on its ability to form polarons, cation radicals [4]. The four double bonds constituting the quinonediimine unit in PANI emeraldine salt convert to three double bonds in a benzene ring and two unpaired electrons, which act as charge carriers. The polarons can eventually spread over the polymer chain to produce a polaron lattice. The polymerization condition has an important effect on the final electrical properties (the conductivity and dielectric loss) of conductive polymers. In the case of PANI, the most used synthesis is the polymerization initiated by an addition of an oxidant, generally ammonium persulfate. This synthetic route leads to the conductive emeraldine salt of PANI, which is formed by the head-to-tail coupling of the monomers. Various fillers such as Ag nanoparticles [5], Bi_2_Se_3_ nanoplates [6], or thermally reduced graphene [7] can be embedded to improve the properties of PANI.

The thermoelectric properties of PPy may be improved by a controlled synthesis of various nanostructures [8]. Nanotubular-type PPy are synthetized through a chemical polymerization route and treated with various dopants such as hydrochloric acid, p-toluenesulfonic acid monohydrate or tetrabutylammonium hexafluorophosphate, which affect its electrical and thermal properties [9]. Also hybrid PPy thermoelectrics have been prepared such as the PPy nanowire/graphene composite [10], the PPy/multiwalled carbon nanotube composite [11], the PPy/Ag nanocomposite film [12], or the PPy/graphene/PANI nanocomposite with a high thermoelectric power factor [13]. 

Thanks to the content of oxygen functional groups on their surfaces, the multiwalled carbon nanotubes (MWCNTs) and the carbon nanofibers (CNFs) embedded into ethylene-octene-copolymer (EOC) may affect its thermoelectric power [14]. Besides the oxygen groups, thermoelectric properties of carbon nanotubes can be changed by doping with many other electron donors [15].

Waste heat dissipated from homoiothermic human bodies can be readily used as the source of electrical power for polymer thermoelectrics, which can be used as unobtrusive low cost self-powered sensors and integrated devices for biometric monitoring [16]. Similarly, thermoelectric self-powered temperature sensors based on Te nanowire/poly(3-hexyl thiophene) polymer composite are described in [17]. Alternatively, such thermoelectrics can be used also as a wearable energy harvester turning radiated human body heat into a source of electric energy. The current research progress on flexible thermoelectric devices and conducting polymer thermoelectric materials is reviewed in [18].

In this paper, hybrid thermoelectrics consisting of EOC matrices and embedded pristine or functionalized MWCNTs, carbon nanofibers, and organic PANI and PPy particles were used to form conductive nanostructures with thermoelectric properties, which at the same time had sufficient strength, elasticity, and stability. Their thermoelectric power was measured and discussed in the light of the oxygen content of their inorganic fillers. A practical application of the prepared thermoelectric composites for electricity microgeneration for a self-powered temperature signaling sensor and a self-powered vapor sensor was introduced.

## 2. Materials and Methods

Purified MWCNTs produced by a chemical vapor deposition of acetylene were supplied by Sun Nanotech Co. Ltd., Jiangxi, China. According to the supplier, the nanotube diameter is 10–30 nm, the length 1–10 μm, the purity ~90% and the volume resistivity 0.12 Ωcm. The diameter of individual nanotubes is between 10 and 60 nm (100 measurements) at the average diameter and the standard deviation is 15 ± 6 nm. The nanotube length is from about 0.2 μm up to 3 μm. They consist of 15 to 35 rolled layers of graphene at the interlayer distance of ca 0.35 nm [19]. The pristine nanotubes are denoted further on as MWCNT(Sun)s.

MWCNTs (BAYTUBES C70 P) were supplied by Bayer MaterialScience AG, Leverkusen, Germany. The nanotube purity is >95 wt %, outer mean diameter ~13 nm, inner mean diameter ~4 nm, length > 1 µm and declared bulk density of MWCNT of agglomerates of micrometric size 45–95 kg/m^3^. The pristine nanotubes are denoted further on as MWCNT(Bayer)s.

The carbon nanofibers (CNFs) with trade name VGCF^®^ (Vapor Grown Carbon Fibers) were supplied by Showa Denko K.K., Tokyo, Japan. The fiber diameter was 150 nm, length 10 μm and electrical resistivity 0.012 Ωcm.

The oxygenated MWCNTs were prepared in a glass reactor with a reflux condenser filled with 250 mL of 0.5 M H_2_SO_4_, into which 5 g KMnO_4_ and 2 g MWCNTs were added. Then the dispersion was sonicated at 85 °C for 15 h using a thermostatic ultrasonic bath (Bandelin electronic DT 103H, Merck spol. s r. o., Prague, Czech Republic). Thereafter, the product was filtered, washed with concentrated HCl to remove MnO_2_, thoroughly rinsed with deionized water and dried [19]. Alternatively, the MWCNTs were oxygenated by HNO_3_ as follows: 2 g of the MWCNTs were added to 250 mL of HNO_3_ (concentrated) and heated at 140 °C for 2 h. After that, the dispersion was cooled and filtered. The sediment was washed by deionized water and dried at 40 °C for 24 h. The corresponding oxygenated MWCNTs are denoted MWCNT(Sun)H_2_SO_4_ or MWCNT(Sun)HNO_3_ and MWCNT(Bayer)H_2_SO_4_ or MWCNT(Sun)HNO_3_.

For preparing PANI emeraldine salt, 0.2 M aniline hydrochloride was mixed with 0.25 M ammonium persulfate (APS) in water, briefly stirred, and left to polymerize for 24 h at room temperature. Then the PANI precipitate was collected on a filter and washed with 0.2 M HCl and acetone. Polyaniline emeraldine particles were in turn dried in air and then under vacuum at 60 °C for 24 h [20].

Pyrrole monomer (Py, purity ≥ 98%, Sigma–Aldrich Inc., St. Louis, MO, USA) was distilled twice under reduced pressure and stored below 4 °C. Polypyrrole particles were prepared via in situ polymerization of the precooled Py in a system containing surfactant CTAB. The precooled initiator APS was added into the system dropwise and the polymerization was allowed to proceed with stirring for 2 h at 0–5 °C. After being washed with water and ethanol, the PPy powder was dried in air and then under vacuum at 60 °C for 24 h. After drying, both types of polymers were gently ground with mortar and pestle.

The reagents APS (purity = 98%) and HCl (concentration ≈ 35%) were purchased from Sigma– Aldrich Inc., St. Louis, MO, USA. The aniline hydrochloride (purity ≥ 99%) was purchased from Fluka, Buchs, Switzerland, cetyltrimethylammonium bromide (CTAB, purity = 98%) from Lach–Ner Ltd., Neratovice, Czech Republic, acetone and ethanol from Penta Ltd., Chrudim, Czech Republic. The reagents were used without further purification.

The carbon allotrope/EOC composites were prepared by an ultrasonication of dispersions of MWCNTs or CNFs in EOC/toluene solution (5% of EOC in toluene). The chosen concentration of the filler in the composites was 30 wt %, which was well above the percolation threshold. The ultrasonication of the dispersion was done in the thermostatic ultrasonic bath (Bandelin electronic DT 103H, Merck spol. s r. o., Prague, Czech Republic) for 3 h at 80 °C. Then the dispersion was poured into acetone to form a precipitate. The final composite sheets were prepared by compression molding at 100 °C [14]. The organic PANI (PPy)/EOC composites were prepared in the same way as the carbon allotrope composites except for filler concentration, which was 70 wt %.

MWCNTs were analyzed by means of transmission electron microscopy (TEM) using the microscope JEOL JEM 2010 (Jeol Ltd., Freising, Germany) at an accelerating voltage of 160 kV. The sample, the MWCNT dispersion in acetone prepared by ultrasonication, was deposited on a 300 mesh copper grid with a carbon film (SPI, Washington, DC, USA) and dried. The structure of CNFs was observed by means of a scanning electron microscope (SEM) Nova NanoSEM 450, FEI, Lincoln, NE, USA, at operating voltage 10 kV. The sample, CNF dispersion in acetone prepared by ultrasonication, was deposited on carbon targets and covered with a thin Au/Pd layer. For the observations, a regime of secondary electrons was used. The same scanning electron microscope was used to observe the morphology of PANI emeraldine salt and of PPy particles.

The X-ray photoelectron spectroscopy (XPS) signals were measured to obtain information on functional groups attached onto the nanotube surfaces. The XPS signals from MWCNT(Sun), MWCNT(Sun)KMnO_4_ and MWCNT(Sun)HNO_3_ network surfaces were recorded using the Thermo Scientific K-Alpha System TFA XPS (Physical Electronics Instrument, Chanhassen, MN, USA) equipped with a micro-focused, monochromatic A1 Ka X-ray source (1486.6 eV). An X-ray beam of 400 μm size was used at 6 mA × 12 kV [21]. The spectra were acquired in the constant analyzer energy mode with pass energy of 200 eV for the survey. Narrow regions were collected using the snapshot acquisition mode (150 eV pass energy) enabling rapid collection of data (5 s per region). The narrow region data were as post-processed using the Jansson’s algorithm to remove the analyzer point spread function, which resulted in an improved resolution of the spectra for the peak deconvolution [22]. The concentration of elements was determined from survey spectra by MultiPak v7.3.1 software (Physical Electronics Inc., Chanhassen, MN, USA).

Fourier-transform infrared (FTIR) analyses of MWCNTs, MWCNT(KMnO_4_)s and MWCNT(HNO_3_)s were performed on the FTIR spectrometer Nicolet 6700 (Thermo Scientific, Waltham, MA, USA). The transmission accessory was used for pristine MWCNT(Sun)s, MWCNT(Sun)KMnO_4_ and MWCNT(Sun)HNO_3_ samples in powder form prepared by potassium bromide. FTIR analyses of chemical composition of PANI emeraldine and PPy particles were examined by the above mentioned FTIR spectrometer Nicolet 6700 using the attenuated total reflectance technique with a germanium crystal in the range 600–4000 cm^−1^ at 64 scans per spectrum at 2 cm^−1^ resolution.

**Figure 1:**
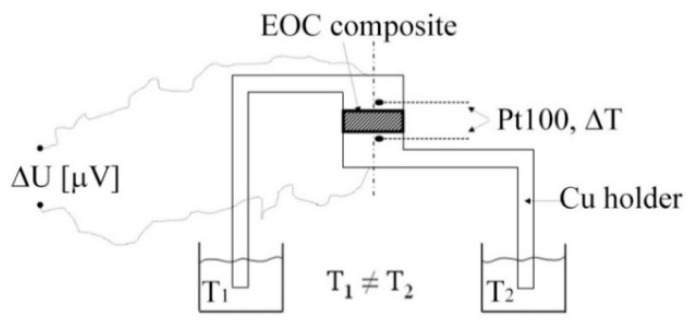
A schematic illustration of the set up for the measurement of electric voltage generated between the hot and cold ends of the Cu/composite/Cu thermoelectric device as a response to a temperature difference.

The Hall coefficient, the resistivity, and the conductivity of the samples as well as the charge mobility and the charge carrier concentration were measured by means of the HCS 1 apparatus (Linseis Messgeräte GmbH, Selb, Germany) equipped with static 0.7 T field permanent magnets for bipolar measurement. The disc form samples had diameter 20 mm and thickness 2.65 mm. The sample current was set to 4 mA. The thermoelectric power measurement was carried out for all the samples using the set-up illustrated in Figure 1. The schematic diagram shows that the circular composite sample (diameter 20 mm, thickness 2 mm) was placed between two copper electrodes. The ends of each of the Cu electrodes were immersed in thermostatic silicone oil baths set at different temperatures. The temperature at the copper/composite interfaces was measured by a Pt100 temperature sensor. The arising thermoelectric current was measured by the Keithley 2000 Digital Multimeter (Tektronix, Inc. Beaverton, OR, USA). 

## 3. Results

### 3.1. Characterization of Fillers and Composites

**Figure 2:**
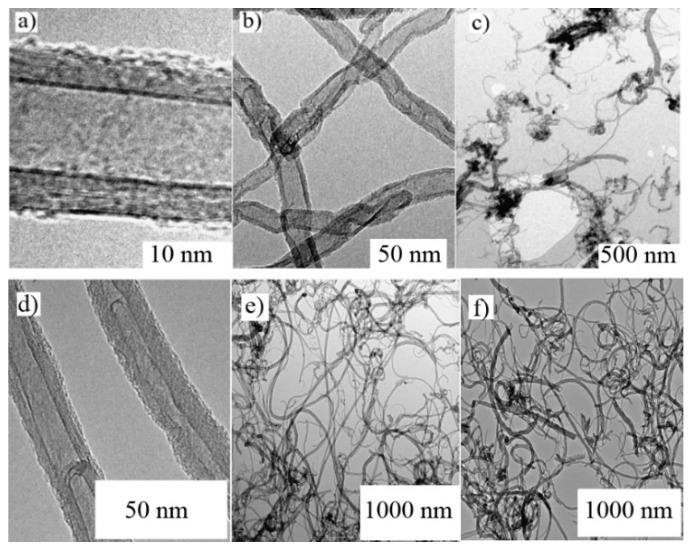
The upper set: Transmission electron microscopy micrographs of MWCNT(Sun)s. (**a**) The structure of an individual pristine nanotube, (**b**) the detail of a nanotube crossing, (**c**) the cluster of pristine nanotubes. The lower set: TEM micrographs of MWCNT(Bayer)s. (**d**) The structure of an individual pristine nanotube, (**e**) the cluster of pristine nanotubes, (**f**) the cluster of oxidized MWCNT(Bayer)KMnO_4_.

The detailed view of individual pristine MWCNT(Bayer)s, their clusters as well as clusters of the oxidized MWCNT(Bayer)KMnO_4_ as obtained by the TEM are shown in Figure 2. The wall of the MWCNT(Bayer) consisted of about 15 rolled layers of graphene. The nanotube outer and inner diameter was about 20 nm and 4–10 nm, respectively. There were also defects obstructing nanotube interiors, which were commonly seen in the MWCNT structures. When a cluster of pristine MWCNT(Sun)s was compared with a cluster of wet oxidized nanotubes, a difference in the tube length was visible. The oxidation of MWCNT(Sun)s by KMnO_4_ caused shortening of the nanotubes, creation of defect sites, and opened ends. A small amount of amorphous carbon after the KMnO_4_ oxidation process can be also expected [23], although another report showed that oxygenation by KMnO_4_ in an acidic suspension provides nanotubes free of amorphous carbon [24]. 

**Figure 3:**
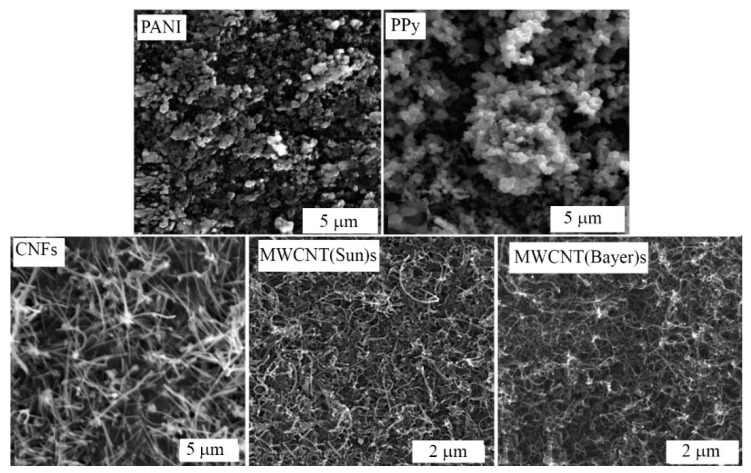
SEM micrographs of a surface of layers made of organic and inorganic fillers. The respective fillers are denoted in the images.

The micrographs of the surfaces of filler layers—PANI and PPy particles as well as MWCNTs and CNFs—are shown in Figure 3. The layers were formed from the respective aqueous dispersion of the fillers on the surface of the interdigitated electrode by the drop method. The particles were globular with a diameter 0.5–1 μm and the PPy particles had narrower size distribution.

The networks of CNFs, MWCNT(Sun)s and MWCNT(Bayer)s resulted from the filtration of their dispersions through non-woven polyurethane membranes. The dispersion, which consisted of CNFs (0.8 mg), or the same amount of nanotubes, dispersed in 530 mL of water with 15.4 g of the surfactant (sodium dodecyl sulphate) and 8.5 mL of the co-surfactant (1-pentanol), was properly sonicated and the pH adjusted to 10 using an aqueous solution of NaOH. The filtrate layer was washed in situ several times with deionized water and finally with methanol.

**Figure 4:**
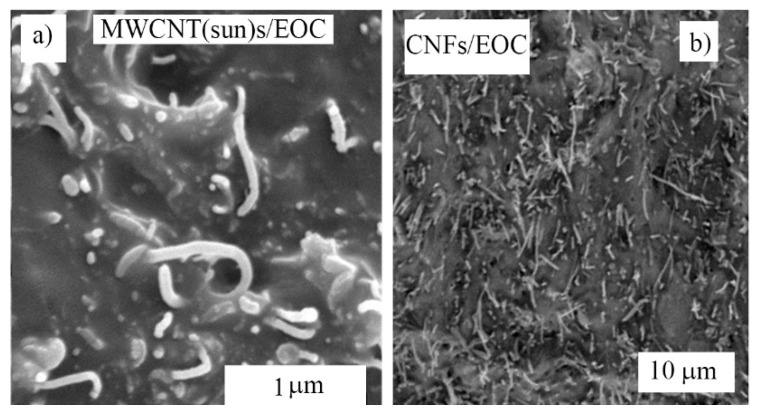
SEM images of cross-sections of carbon allotrope/ethylene-octene-copolymer matrices (EOC) composites. The respective composites are denoted in the images.

The cross-section of the composites presented in Figure 4 showed a uniform distribution of the MWCNT(Sun) and CNF filler in the EOC matrix, which together with the filler concentration 30% ensured the electrical and thermal conductivity of the composites.

### 3.2. XPS Data

**Figure 5:**
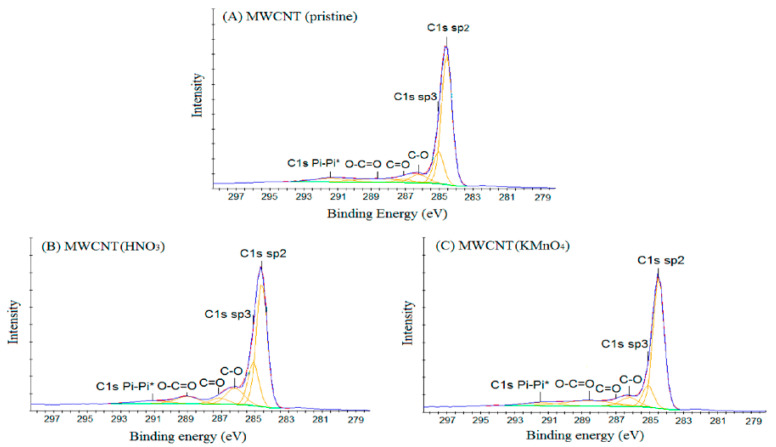
XPS C1s spectra for MWCNT(pristine) (**A**), MWCNT(HNO_3_) (**B**) and MWCNT (KMnO_4_) (**C**) samples.

The main binding energy peak (284.5 eV) in the XPS spectra of pristine MWCNT(Sun)s, Figure 5, was assigned to the C1s-sp2, while the other ones were assigned to the C–O (286.2 eV), C=O (287.1 eV), O–C=O (288.6–289 eV) and C1s-π-π* (291.1–291.5 eV). After the oxidation treatment, the intensities of the peaks corresponding to the oxidized carbon bonds increased as seen in Figure 5. FTIR data of the MWCNTs also confirmed the presence of the C–O, C=O and O–C=O functional groups on their surfaces. It is also stated in other studies that MWCNTs treated with (NH_4_)_2_S_2_O_8_, H_2_O_2_, or O_3_ have higher concentrations of carbonyl and hydroxyl functional groups, while more aggressive oxidants (HNO_3_, KMnO_4_) form higher fractional concentrations of carboxyl groups [25]. Acidic potassium permanganate (KMnO_4_) is a strong oxidizing agent and produces more surface acidic groups than nitric acid [26]. The increase number of oxygenated functional groups attached on MWCNT(Sun)KMnO_4_ surfaces significantly increases the contact resistance in the MWCNT junctions of the network structure [27].

### 3.3. FTIR Measurements

**Figure 6:**
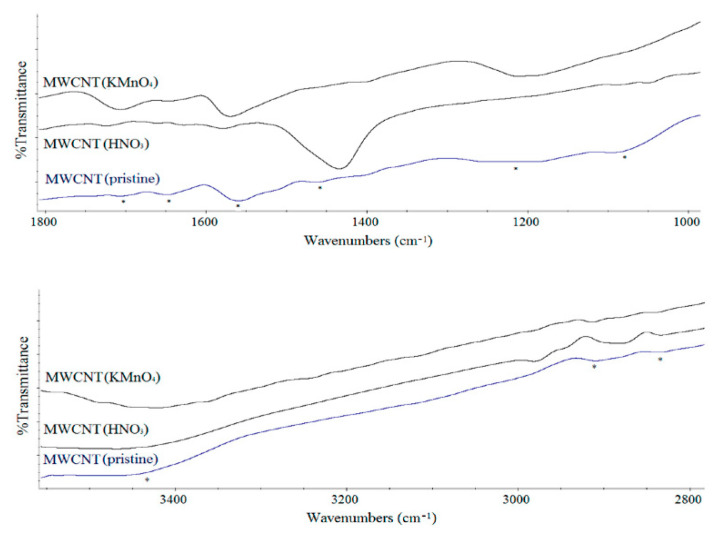
FTIR spectra of the MWCNT samples in the range of 1000–1800 cm^−1^ and 2800–3500 cm^−1^.

Table 1 and Figure 6 present the frequencies of some of the functional groups in the FTIR spectra of MWCNT networks. FTIR spectra in Figure 6 from MWCNTs showed a broad peak about 3430 cm^−1^ which was characteristic of the O–H stretch of hydroxyl group. C–H stretching of the pristine MWCNT(Sun) sample was shifted to lower wavelengths for all oxidation treatments. A weak C=O peak at 1705 cm^−1^, Figure 6, was observed in the pristine MWCNT(Sun) sample, which showed that there was a carbonyl or carboxylic group on its surface. The reason why the pristine MWCNT(Sun) sample had carbonyl and OH groups could be a partial oxidation of the surfaces of MWCNTs during the purification by the manufacturer [28]. A higher shift in the carbonyl stretching mode was seen for MWCNT(HNO_3_) than for MWCNT(KMnO_4_). The reason could be a C=O group or other groups that interacted with the C=O group. FTIR spectra in Figure 6 also indicated that there were probably no anhydride/lactone groups on the surfaces of MWCNTs since these groups are usually observed at around 1750 cm^−1^ or higher wavenumber [26,29,30].

The peak assigned to the quinone group at 1652 cm^−1^ in MWCNT(pristine) sample was usually shifted to a higher wavelength in oxidized MWCNTs. Coupling effects (i.e., both of inter-molecular and intra-molecular hydrogen bonding with hydroxyl groups) also might be responsible for the downshift in the C=O stretching mode, besides the production of surface-bound quinone groups with extended conjugation [31].

The up-shift in the C=C stretching mode of MWCNTs was observed for both oxidized carbon nanotubes. The highest shift was observed for MWCNT(HNO_3_) compared to MWCNT(KMnO_4_). This treatment suggested a change in the structure of the MWCNTs [32].

The C–H (CH_2_/CH_3_) bending at 1460 cm^−1^ and the peak at 1222 cm^−1^ for the MWCNT(pristine) sample were shifted to a higher wavelength for all oxidized MWCNTs. There was also observed a new band around 1050 cm^−1^ in the FTIR spectra of oxidized MWCNTs, which could be assigned to the alcoholic C–O stretching vibration [33]. Overall, the observed changes in the FTIR spectra of the oxidized MWCNTs confirmed the efficiency of the oxidizing process and the formation of the new oxygen-containing functional groups on the surfaces of the carbon nanotubes.

**Figure 7:**
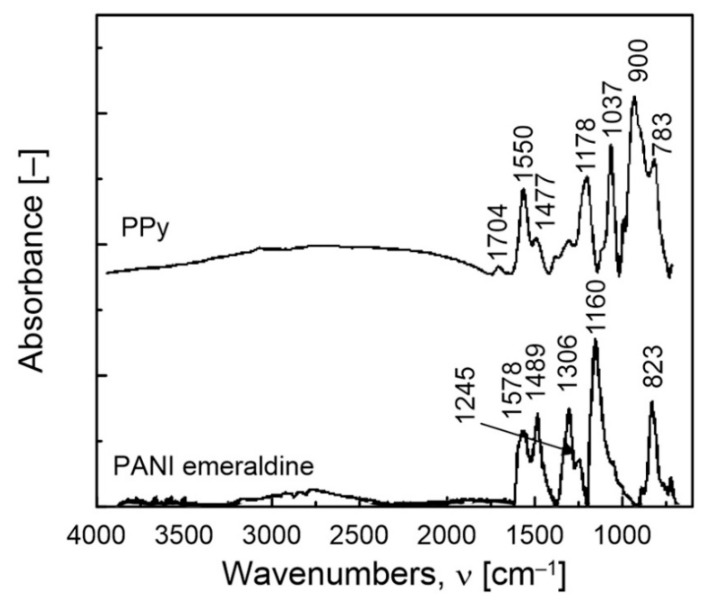
FTIR spectra of the PANI emeraldine and PPy particles.

FTIR analysis was also carried out to identify whether the prepared powder was indeed PANI emeraldine or PPy (Figure 7). For the FTIR absorption spectroscopy of the prepared PANI emeraldine, the vibration at 1578 cm^−1^ was attributed to the quinoid ring, while the vibration at 1489 cm^−1^ depicted the presence of a benzoid ring unit [34]. The peak at 1306 cm^−1^ is assigned to the C–N stretching of a secondary aromatic amine. Furthermore, the peak characteristic for the electrically conductive form of PANI emeraldine was observed at 1245 cm^−1^. This peak was attributed to the stretching of the C–N^+•^ polaron structure [35]. The peak at 1160 cm^−1^ corresponding to the vibrations associated with the C–H of N=Q=N (Q = quinoid ring) also appeared in the spectrum of PANI emeraldine. The band between 913–680 cm^−1^ with a maximum at 823 cm^−1^ was characteristic for an aromatic ring deformation and C–H bond vibrations out of the plane of the ring [36]. These results suggested that the synthesized particles were PANI in the emeraldine state. For the FTIR spectroscopy of the prepared PPy, all the characteristic peaks were in good agreement with the earlier investigations of the same product [37,38]. The vibration peak at 1704 cm^−1^ was assigned to the C=N bond. Peaks at 1550 and 1477 cm^−1^ attributed to the in-plane vibrations of the PPy ring, and the peaks at 1178, 1037 and 783 cm^−1^ attributed to the in-plane bending of the PPy ring were also observed. Finally, the characteristic peak at 900 cm^−1^ was assigned to the out-of-plane vibration of the C_β_–H group.

The experimental test of the electric transport properties of the MWCNT(Sun)pristine/EOC composite at room temperature specified the following values: the sample resistance 0.87 Ω, the resistivity 0.23 Ωcm, the conductivity 4.3 Ω^−1^cm^−1^ and the magneto-resistance 0.249 mΩ. Further determined properties were the Hall-mobility 7.2 cm^2^/Vs, the charge carrier concentration (bulk) 3.7 × 10^18^ cm^−3^ and the average Hall coefficient +1.67 cm^3^/C. The positive sign of the Hall coefficient identified the nature of the composite, i.e., the p-type of drift current when the holes are the dominant current carriers.

### 3.4. Thermoelectric Power Measurement

**Figure 8:**
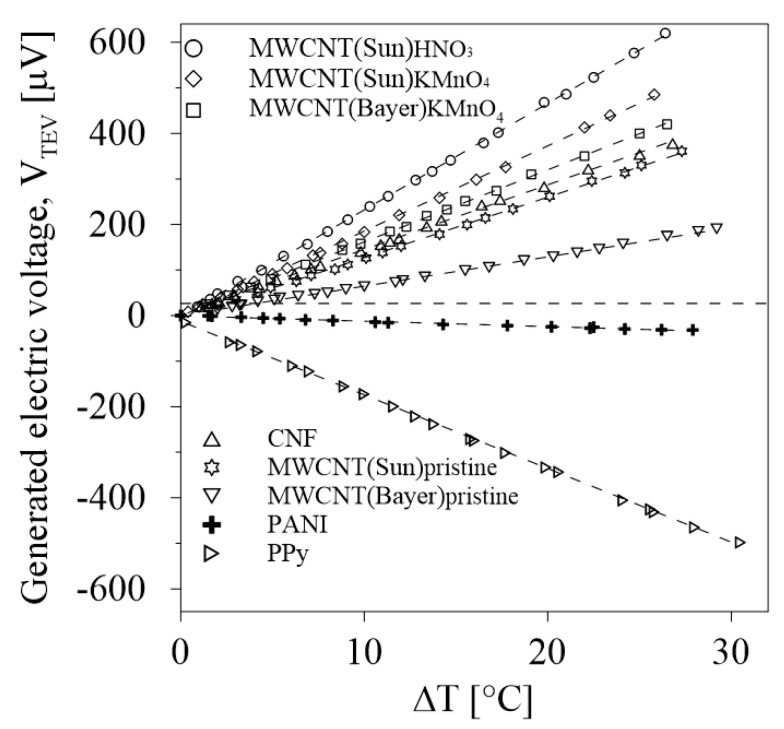
The relations between generated voltage and temperature difference for all investigated EOC composites.

**Figure 9:**
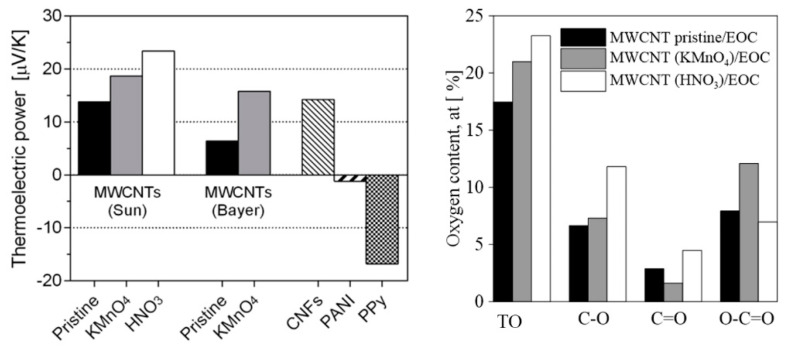
Thermoelectric power for tested EOC composites with indicated embedded fillers (**left panel**). Oxygen content (%) on surfaces of pristine and differently oxygenated MWCNTs (**right panel**). TO denotes the total oxygen amount and the C–O, C=O, and O–C=O the amount of the particular functional groups on the surface of the respective MWCNTs.

Values of induced voltage in response to a temperature difference across the measured samples of the investigated thermoelectric composites are presented in Figure 8. The slope of the linear dependence of the resulting voltage V_TEV_ on the temperature difference Δ*T* defined the thermoelectric power (Seebeck coefficient):

$$S={\left(V_{\mathrm{TEV}}/\Delta T\right)}_{\mathrm{open}\;\mathrm{circuit}}$$

which evaluated a potential thermoelectric performance. The corresponding thermoelectric power values are summarized in Figure 9. The thermoelectric power of MWCNT/EOC composites was substantially enhanced by the nanotube oxygenation. When compared to the composite with pristine nanotubes (MWCNT(Bayer)pristine/EOC), the respective composite with oxygenated nanotubes achieved a 150% increase of thermoelectric power. 

The X-ray photoelectron spectroscopy (XPS) was performed on MWCNTs to ascertain the functional groups attached onto the nanotube surfaces. The oxygen contents (%) of the pristine and oxidized MWCNT samples, as calculated from the XPS spectra, are shown in Figure 9. The comparison of results indicated that all MWCNTs have C–O, C=O and O–C=O groups on their surfaces and that MWCNT(HNO_3_)s have the maximum percentage of all the oxygen-containing groups. Moreover, the more oxygenated functional groups at the surface of the embedded MWCNTs, the higher the generated electric voltage and the thermoelectric power of the EOC composite as follows from the results in Figure 9. According to the published results on carbon nanotube/thermoplastic polymer composites, positive thermoelectric powers have always been determined [39].

### 3.5. Self-Powered Signaling Sensor of Temperature Change

**Figure 10:**
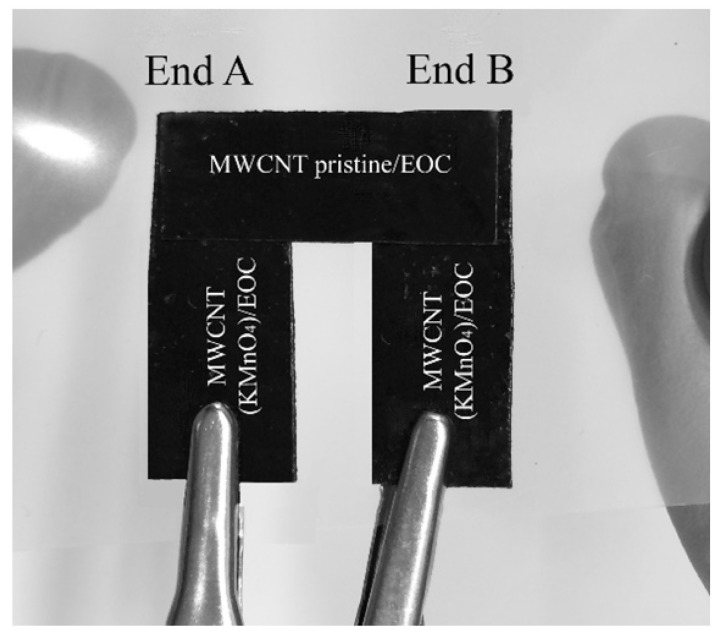
The self-powered signaling sensor of temperature change assembled from MWCNT(Sun)pristine/EOC and MWCNT(Sn)KMnO4/EOC composites.

**Figure 11:**
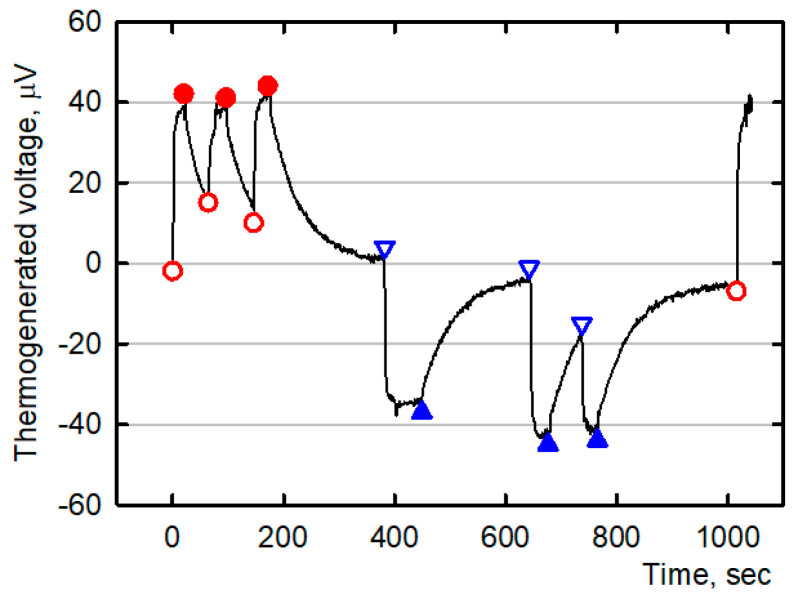
The illustrative time-dependent record of induced voltage in the self-powered signaling sensor by short finger touching at the point A (the finger touch is denoted by open red circles and the finger lift by filled red circles) or at the point B (the finger touch is denoted by open blue triangles and the finger lift by filled blue triangles) of the sensor.

To demonstrate a possible use of the conductive MWCNT/EOC composites as thermoelectrical materials, a self-powered signaling sensor of temperature change was assembled. The sensor consisted of three conductive strips (thermoelements): one MWCNT(Sun)pristine/EOC and two MWCNT(Sun)KMnO_4_/EOC composites, Figure 10. The sticky strips were stuck on a PET foil so that the ends overlapped at points A and B. The temperature signal was induced by a finger touch at point A or B, which in turn heated the connection of the strips. The generated electricity was monitored by the Multiplex datalogger 34980A. Even a temperature gradient induced by a mere short touch of a finger sufficed to elicit a detectable signal. The illustrative record of repeated finger touches is presented in Figure 11. Heat transfer from the finger to the hot junction of the sensor modeled a technological situation when such a sensor could monitor for example changes in the generation of waste heat, changes of technological temperature, warming of packaged grocery products, etc. 

### 3.6. Self-Powered Vapor Sensor

**Figure 12:**
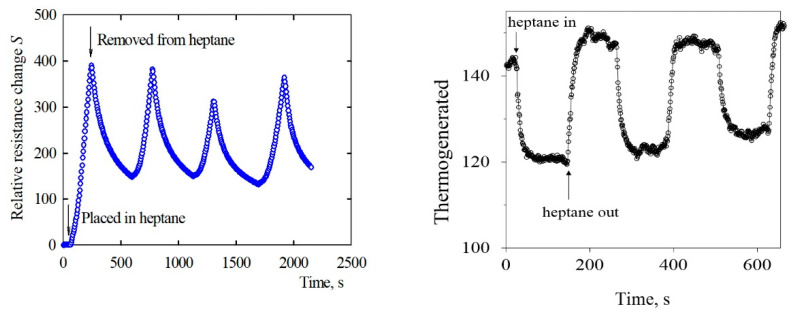
The time-dependent relative resistance change of the MWCNT(Sun)pristine/EOC composite (in %) during adsorption/desorption cycles of exposition to saturated vapors of heptane at 22 °C, the cycle length 600 s. The changes of the thermogenerated voltage induced by the self-powered vapor sensor in the course of three heptane adsorption and desorption cycles at 22 °C.

The induced voltage in the self-powered sensor (Figure 10) was changed not only by heating, but also by ambient organic vapors, Figure 12. As in our paper [14], the self-powered sensor was exposed to saturated organic solvent vapors. In particular, the sensor was placed in a glass bell, which enclosed a layer of liquid organic solvent and its respective saturated vapor. After a chosen time, the sensor was removed from the bell and the effect of vapor desorption on induced voltage was assessed. Such measurements were conducted in saturated vapors at atmospheric pressure, temperature 22 °C, and relative humidity 60%. The temperature gradient in the sensor was induced by means of the resistive heating of the MWCNT network/epoxy composite unit placed under the thermoelement junction. The heating DC voltage was 13 V and the current 0.09 A. The temperature of the hot end was 40.6 °C and of the cold end ambient room temperature. 

The variations of the voltage of the self-powered sensor were caused by the changes of the electric resistance of the forming EOC composites through the chemical signals during the respective adsorption/desorption cycles as is illustrated in Figure 9. When the sensor was subjected to heptane, a larger increase of relative resistance resulted in a larger decrease of induced voltage and vice versa. Thus, the sensor output in terms of variation of voltage indicated a good response to the vapor occurrence. Moreover, considering that the sensor did not require a power supply, but it self-produced thermoelectricity from a heat source, which could be waste heat of industrial processes, solar energy, or body heat, etc., then the self-powered vapor sensor appears to be an advantageous alternative to powered vapor sensors. The illustrative thermoelement resistance changes plotted in Figure 10 were quantified by the relative resistance change,

$$S=\frac{R_{g}-R_{a}}{R_{a}}=\frac{\Delta R}{R_{a}}$$

where *R_a_* represented the initial thermoelement resistance in the air, *R_g_* the resistance when the thermoelement was exposed to a vapor, and Δ*R* denoted the measured resistance change.

## 4. Discussion and Conclusions

This study presents hybrid thermoelectric composites consisting of organic EOC matrices and embedded conductive inorganic pristine and functionalized multiwalled carbon nanotubes, carbon nanofibers or conductive organic PANI and PPy particles. All these composites induced voltage in response to a temperature difference across the respective samples. This response was affected in the case of the composites with inorganic MWCNT nanostructures by the amount of oxygen containing functional groups attached to their surfaces. The more oxygenated were the functional groups at the surfaces of the embedded MWCNTs, the higher was the thermoelectric power of that EOC composite.

According to the published results about carbon nanotube/thermoplastic polymer composites, positive thermoelectric powers have always been determined indicating *p*-type composites, in which holes are dominant current carriers. The composites with embedded organic PANI a PPy particles induce compensating negative voltage. According to [38], the composites exhibit *n-*type conductivity specified by dominating electrons as charge carriers.

The chosen combination of the two *p-*type EOC composites in the experimental thermoelectric microgenerator, which generated sufficient electric current to make the sensors self-powered, was one of many other possible combinations. Other combinations such as *p-*type together with *n-*type thermoelements or two *n-*type EOC composites could have been chosen as well. An illustration of the practical use of the EOC composites as a thermoelectric generator was provided by the self-powered signaling sensor of temperature change and the self-powered vapor sensor. The voltage induced in the sensors, which was in the range of microvolts, did not approach by far the magnitude of induced voltage in the classical metal thermocouples, yet it was sufficient to power the novel sensor device, which converted chemical signals to electric ones. In the self-powered vapor sensor, the resistance varied according to the ambient chemical signals and changed the voltage induced by a source of (waste) heat. The EOC thermoelectric composites thus offer a novel and unique set of properties, which are not readily available in any other material.

## Figures and Tables

**Table 1:** Summary of FTIR measurements for the pristine and oxidized MWCNT networks.

	Wavenumber (cm^−1^)		
Possible Assignments	MWCNT	MWCNT	MWCNT
		(HNO_3_)	(KMnO_4_)
OH stretch	3435	3428	3427
C–H stretch (CH_2_, CH_3_)	2908,2840	2980,2880	2978,2890
C=O stretch (carboxyl or ketone)	1705	1726	1710
Intermediate oxidized products—quinone groups	1652	1661,1635	1641
C=C stretch	1559	1580	1569
CH_2_/CH_3_ bending	1460	1437	1440
Skeletal C-C tangential motions +C–O stretch	1222	1184	1190
C–O stretch	1082	1084,1049	1087,1046
